# Hemoadsorption in LVAD Surgery: Suitable in Theory?

**DOI:** 10.3390/jcdd10070286

**Published:** 2023-07-05

**Authors:** Mahmut Ozturk, Aron Frederik Popov

**Affiliations:** 1 Division of Cardiac Surgery, Children’s National Hospital, School of Medicine and Health Sciences, The George Washington University, Washington, DC 20010, USA; 2 Department of Cardiothoracic Surgery, Hannover Medical School, 30625 Hannover, Germany

The incidence of patients with heart failure is growing steadily, particularly due to advancements in medical therapy. Even the sickest patients with cardiac defects that were once uniformly fatal can now be palliated well into adulthood and are expected to contribute to this growth [1]. While heart transplantation remains the gold standard therapy in patients with end-stage heart failure (HF), mechanical circulatory assist devices grant the prospect of alleviating the burden of limitations in organ availability.

Today, left ventricular assist devices (LVADs) encompass a broad spectrum of therapy alternatives from bridge-to-transplantation to bridge-to-recovery and can be implanted with minimally invasive techniques [2]. Nevertheless, a substantial portion of patients undergoing LVAD surgery still face adverse events such as right heart failure, multi-organ failure, an increased risk of bleeding and sepsis, and marked vasoplegia, which account for increased morbidity and mortality [3,4,5,6]. An exaggerated systemic inflammatory response is hypothesized to play a significant role in the development of multi-organ failure and other adverse events in LVAD patients [7,8]. However, neither an exact pathomechanism nor potential markers to predict the extent of postoperative inflammatory response have been delineated. It has been suggested that higher preimplantation interleukin (IL)-6 levels correlate with worse preimplantation Interagency Registry for Mechanically Assisted Circulatory Support (INTERMACS) profiles and attenuated expression of other monocyte-related inflammatory mediators, longer hospital stays, and worse clinical outcomes after implantation [7]. IL-6, IL-8, and neopterin levels increase after LVAD implantation, especially in those with postoperative multi-organ failure, which is the leading cause of death in patients with mechanical circulatory support, and early elevation in these markers postoperatively is associated with higher mortality [7,8]. Similar to IL-6, tumor necrosis factor alpha (TNF-a) is elevated in chronic HF patients and correlates with the severity of disease and prognosis and is involved in angiodysplasia through pericyte apoptosis and angiopoetin-1 suppression [9,10]. In light of the current findings, the significance of inflammatory cytokines and dysregulated inflammatory response in postoperative course and outcomes for patients undergoing LVAD surgery appears noteworthy.

In recent years, there has been a growing interest in the utilization of extracorporeal hemoadsorption techniques in cardiac surgery to mitigate the burden of dysregulated immune response by immunmodulation through the elimination of cytokines from the systemic circulation [11,12,13,14]. In line with its suggested efficacy in sepsis [15], hemoadsorption therapy has yielded promising results in patients undergoing cardiac surgery, especially in patients with acute infective endocarditis or those with elevated proinflammatory cytokine levels prior to surgery [14,16,17]. Despite relatively small cohort sizes, a major reduction in circulating levels of IL-6, IL-8, bilirubin, lactate, and lactate dehydrogenase have been observed postoperatively in different studies. Correspondingly, improved hemodynamic stability with the reduced need for vasopressors and improved early mortality rates have been reported [14,17,18,19].

In spite of the similar characteristics of end-stage heart failure or LVAD patients and patients with active infection in terms of elevated proinflammatory cytokines, elevated thrombosis and bleeding risk, hepatic and renal dysfunction, angiodysplasia, and clinical frailty with worse operative outcomes, the adoption of hemoadsorption filters at the time of LVAD surgery or during intensive care stay as an adjunct therapy to hemofiltration is limited. Besides individual clinical experience, our understanding of the potential role of hemoadsorption filters in LVAD implantation depends on, if not one, a few studies. In Zhigalov and associates’ single-institution study, in which they compared a treatment group to a matched group, patients treated with a new hemoadsorption filter (Cytosorb-Cytosorbents Europe GmbH) did not benefit in terms of survival and they were more likely to develop respiratory failure (54% vs. 30%), require mechanical ventilation for longer than 6 days (50% vs. 28%), and require more tracheostomies (31% vs. 13%) [20]. In light of their findings, they interpreted that Cytosorb hemoadsorption filters might not offer a substantial morbidity or survival benefit for patients undergoing LVAD implantation. However, despite propensity matching, patients treated with Cytosorb had higher preoperative INTERMACS profiles and IL-6 levels as well as more preoperative ventilation and concomitant surgeries. Furthermore, in this study, the average cardiopulmonary bypass time for the therapy group was 76 min in contrast to the producer’s suggestion of 120 min.

An association between the use of hemoadsorption in cardiogenic shock patients during LVAD surgery and postoperative vasopressor support, improved hemodynamics, or accelerated lactate clearance was also not observed by Pausch and associates [21]. Moreover, the 30-day mortality rate was significantly higher in their hemoadsorption group.

On the other hand, in their recent retrospective study, Haidari and associates analyzed the impact of the intraoperative use of hemoadsorption in heart failure patients undergoing LVAD implantation with cardiopulmonary bypass and reported a significant reduction in postoperative vasoplegia incidence in the treatment group. A significant early mortality difference was not observed between the matched groups with similar INTERMACS characteristics, though both the 30- and 90-day mortality rates were lower in the treatment group (11% vs. 19% (*p* = 0.18) and 23% vs. 29% (*p* = 0.37)) [19].

The ongoing randomized clinical trial Cytosorb Modulation of Surgical Inflammation During LVAD Insertion (CYCLONE-LVAD) intends to investigate the efficacy of Cytosorb treatment during LVAD implantation in the mitigation of postoperative vasoplegia and organ dysfunction as well as perioperative changes in IL-6 [22]. While the initial results have not yet been published, this trial will surely contribute to our understanding of hemoadsorption during LVAD surgery and will potentially provide references for the estimation of postoperative course and outcomes for individual patients.

Although the initiation of LVAD therapy has changed the fate of patients with end-stage heart failure, these patients still represent an extremely fragile population. In addition to fine-tuning the surgical technique, further research aimed at defining potential biomarkers in LVAD patients and the benefit of eliminating them from the systemic circulation through hemoadsorption is warranted. In consideration of the efficacy of cytosorbents in patients with active endocarditis or sepsis and its declared capacity to filter out cytokines such as IL-6, TNF-a, and interferon-gamma or anticoagulant drugs such as P2Y12 inhibitors or factor Xa inhibitors, its safety and feasibility in patients undergoing surgery with (a) myocarditis or active endocarditis, (b) severe hepatic or renal dysfunction and hyperbilirubinemia, and (c) in urgent need of cardiac surgery despite effective anticoagulation therapy need to be further investigated. The impact of proinflammatory cytokine filtration at the time of both LVAD and heart transplantation surgeries on the reduction of organ rejection also bears substantial potential for groundbreaking research and deserves further attention.
